# Family Financial Pressure in Childhood and Telomere Length in Early Adolescence: A Prospective Study

**DOI:** 10.3390/genes13050721

**Published:** 2022-04-20

**Authors:** Keith T. S. Tung, Rosa S. Wong, Hing Wai Tsang, Wilfred H. S. Wong, Winnie W. Y. Tso, Jason C. Yam, Terry Y. S. Lum, Godfrey C. F. Chan, Ian C. K. Wong, Patrick Ip

**Affiliations:** 1 Department of Paediatrics and Adolescent Medicine, The University of Hong Kong, Hong Kong, China; keith-tung@connect.hku.hk (K.T.S.T.); thwpaed@hku.hk (H.W.T.); whswong@hku.hk (W.H.S.W.); wytso@hku.hk (W.W.Y.T.); gcfchan@hku.hk (G.C.F.C.); 2 Department of Social Work and Social Administration, The University of Hong Kong, Hong Kong, China; tlum@hku.hk; 3 Department of Pharmacology and Pharmacy, The University of Hong Kong, Hong Kong, China; wongick@hku.hk; 4 State Key Laboratory of Brain and Cognitive Sciences, The University of Hong Kong, Hong Kong, China; 5 Department of Ophthalmology and Visual Sciences, The Chinese University of Hong Kong, Hong Kong, China; yamcheuksing@cuhk.edu.hk; 6 Research Department of Practice and Policy, UCL School of Pharmacy, University College London, London WC1N 1AX, UK

**Keywords:** telomere, early childhood exposure, family financial pressure

## Abstract

Much research on children in high-risk environments has focused on the biological consequences of maltreatment, adversity, and trauma. Whether other early-life stress sources such as family financial hardship are implicated in the cellular mechanism of disease development remains unclear. This study investigated the long-term effect of childhood exposure to family financial pressure on telomere length. It involved two waves of data collection occurring when participants reached Grade 3 (W1) and 7 (W2), respectively. In W1, parents reported family demographics and perceived financial stressors and pressure. In W2, participants provided buccal swab samples for measurement of their telomere length. Data from 92 participants (M_age_ in W2 = 13.2 years; 56.5% male) were analyzed. The main type of stressors reported by parents who perceived high family financial pressure in W1 were child-level stressors including affordability of their medical and educational expenses. Participants exposed to high parent-perceived family financial pressure in W1 had shorter telomeres in W2 when compared to those exposed to low parent-perceived family financial pressure (β = −0.61, *p* = 0.042). Subgroup analyses revealed stronger associations in girls than boys. These findings reveal an important spillover effect between parental financial perceptions and stress and children’s health at the cellular level.

## 1. Introduction

Research has indicated that early-life household financial condition is an important determinant of health and development [1]. It has been suggested that objective indicators such as income and wealth are not sufficient to fully capture household financial situation [2,3,4]. On the other hand, subjective indicators such as perception of financial burden can provide reliable information as to whether the respondents consider themselves to be in a difficult financial situation [2]. Although financial issues may not have a direct effect on children, living in a family environment with high financial stress has been found to be associated with poor health and well-being [5]. The family stress model proposes that parenting skills and family dynamics together with other factors contribute to the association between family financial hardship and children’s wellbeing [6]. Specifically, financial hardship might exacerbate family conflicts and increase maladaptive parenting practices [7], which in turn can lead to health and developmental problems in children [8,9]. Evidence also suggests that repeated exposure to traumatic events in early life can contribute to future overreactions to stressors and potentially induce permanent physiological changes, thereby resulting in an increased risk of chronic diseases [10].

Biomarkers such as telomeres have been used to quantify the acute and chronic biological effect of stressful experiences. Telomeres as a biomarker reflecting the rate of aging process are specialized nucleoprotein complexes at the chromosome ends that promote chromosomal stability [11]. Replication flaws can result in gradual telomere shortening, yet this process can be accelerated by cellular damage due to suboptimal health conditions such as physical and psychiatric diseases and traumatic experiences [12,13,14]. For example, a previous study of children aged 8 to 13 years found a strong association between telomere shortening and negative mood resulting from disrupted family interaction [15]. More studies point to the biological consequences of severe early-life stressors including exposure to maltreatment and abuse [15,16]. However, little work has been done to examine the impact of milder forms of family stressors on children’s health and well-being. Some previous studies have found that growing up in low socioeconomic status (SES) families was associated with shorter telomere length (TL) [17,18,19,20], but these studies mainly used objective measures such as parental educational attainment and family income levels to assess household financial condition. No studies to date have examined the association between children’s cellular and molecular responses and parental perception of household financial conditions.

Therefore, this study used two-wave longitudinal data to investigate whether early-life exposure to family financial pressure was associated with shorter TL in early adolescence. The stressor profiles for parents who perceived low family financial pressure, those perceiving population-equivalent family financial pressure, and those with high family financial pressure were investigated. Considering the likelihood of gender differences in cellular responses to stressors [21], we also examined whether the impact of early-life exposure to family financial pressure on later TL would differ by gender. Family income level was another potential effect modifier tested in this study, as the perception of financial pressure is subjective and could be independent of actual income.

## 2. Materials and Methods

### 2.1. Study Design and Participants

This is a sub-study of the Healthy Kids cohort study in Hong Kong that was initiated in 2011–2012 and has undergone two waves of follow-up data collection in 2014–2015 and 2018–2019, respectively, with an aim to examine the long-term health and developmental impact of early-life SES status [22,23]. Data from healthy participants who completed the 2014–2015 (W1) assessment and provided buccal swab samples in the 2018–2019 (W2) assessment were analyzed. Written informed consent from parents/guardians were obtained prior to recruitment, data collection, and biological samples collection.

### 2.2. Measures

#### 2.2.1. Early Adolescence Telomere Length in W2

TL in early adolescence was determined using the buccal swab samples collected in W2. Upon the DNA extraction using QIAamp DNA Mini kit (Qiagen), the quality and quantity of the extracted DNA samples were checked using a Nanodrop 2000c spectrophotometer (Applied Biosystems). DNA samples with an A260/A280 ratio of 1.8 ± 0.1 and a concentration of higher than 5 ng/μL were considered to have satisfactory quality and quantity for TL determination. DNA samples with satisfactory quality and quantity were handled in triplicate to determine the average TL using quantitative polymerase chain reaction according to the procedures described in previous studies [11,24]. To enhance the accuracy of the measurement, inter-plate variability was adjusted by including a control sample in each PCR plate. The TL was expressed as a relative ratio of the telomere repeat copy number (T) to single-copy gene 36B4 copy number (S) with the formula “T/S = 2−ΔCt”, where ΔCt is the mean difference between the threshold cycle (Ct) value of the 36B4 gene and telomere repeats.

#### 2.2.2. Early-Life Family Financial Condition in W1

Early-life family financial condition was measured with two parts of items concerning family financial pressure and parental financial stress, respectively. In W1, parents were asked to report their own family financial pressure level with reference to the level of financial pressure they perceived to be on other families in Hong Kong on a 5-point scale ranging from 1 = none/almost none to 5 = enormous. Based on their responses, participants were categorized into three family financial pressure groups: low (N = 32), population-equivalent (N = 41), and high (N = 19).

In addition, parents completed a 9-item family financial stress scale with items concerning family, personal or child stressors in the past 6 months on a 6-point scale ranging from 1 = totally disagree to 6 = totally agree. Specifically, there were items on family stressors including family financial burden, family activities, and impacts on family conflicts and family relationship; personal stressors including satisfaction with their own financial condition, worry due to financial problems, and financial capacity; and child stressors including the abilities to afford the child’s medical and educational expenses and purchase what the child wants.

#### 2.2.3. Demographics Characteristics

In W1, parents reported their employment and marital status, monthly family income, and status of Comprehensive Social Security Assistance (CSSA) (government financial assistance scheme for poor families) as well as children’s gender and date of birth. All these demographic variables were included as covariates in subsequent analyses.

### 2.3. Data Analysis

All analyses were conducted using the SPSS Statistics (version 26.0). Descriptive statistics were first generated to describe sample characteristics. One-way analysis of variance (ANOVA) or Kruskal-Wallis test (for continuous variables) and chi-square analyses (for categorical variables) were conducted to examine whether sociodemographic characteristics and TL in W2 differed between participant groups based on their level of exposure to parent-perceived family financial pressure in W1.

Linear regression analyses were then performed to evaluate the associations between parent-perceived family financial pressure and their level of agreement on stressors at the family, personal and child level. Linear regression models were also conducted to examine the effects of parent-perceived family financial pressure (measured in W1) on participants’ TL (measured in W2). These regression models were all adjusted for children’s age and gender and W1 socioeconomic conditions (family income level and parental marital status and employment status). To examine changes in the strength of associations, parent-perceived family financial pressure was examined as both categorical and continuous variables in the regression models. Subgroup analyses were performed to examine the effect of parent-perceived family financial pressure in W1 on participants’ TL in W2 by (i) gender (boys vs. girls) and (ii) monthly family income level (below HKD 20,000 (USD 2564, low family income) vs. HKD 20,000 or above (average-to-high family income). We defined family income below HKD 20,000 as low family income, because the Hong Kong median monthly 3-member household income in 2015 was HKD 27,500 (USD 3526) [25]. Due to high skewness, a logarithmic transformation was performed on TL before the analysis. All the TL values after logarithmic transformation were within 3 standard deviations from the group mean. A *p*-value of less than 0.05 indicated statistical significance.

### 2.4. Ethical Approval

The research protocol was approved by the Institutional Review Board of the University of Hong Kong/Hospital Authority Hong Kong West Cluster (Reference: UW 18-057). The authors assert that all procedures contributing to this work comply with the ethical standards of the Institutional Review Board of the University of Hong Kong/Hospital Authority Hong Kong West Cluster on human experimentation and with the Helsinki Declaration of 1975, as revised in 2008.

## 3. Results

### 3.1. Descriptive Statistics

**Figure 1:**
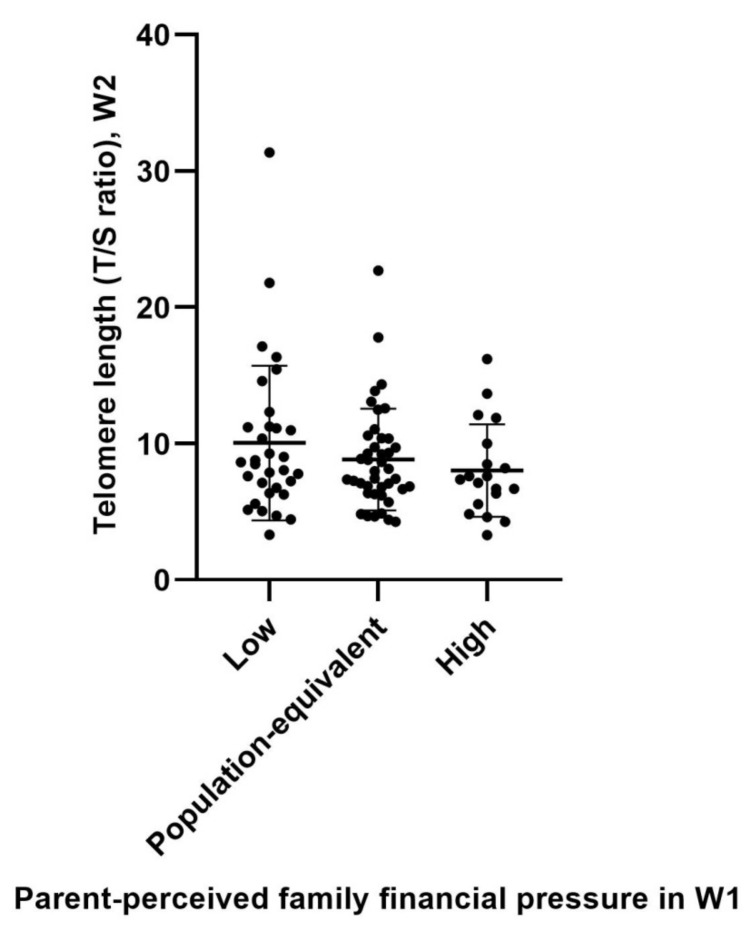
Telomere length in W2 among adolescents by different levels of exposure to parent-perceived family financial pressure in W1.

A total of 92 healthy participants were included in this study. Table 1 shows the sample characteristics overall and by W1 parent-perceived family financial pressure group membership. Among the 92 participants (average age in W2: 13.2 years), 52 (56.5%) were boys and 40 (43.4%) were girls. Over 90% of the fathers and 45% of the mothers worked full-time. The average monthly household income in W1 was HKD 46,209 (USD 5924). 20.7% of the parents perceived themselves to be under a high level of financial pressure when compared to other local families, and these families were more likely to have a single parent and had lower family income level. Their mean TL before logarithmic transformation (T/S ratio) was 9.08 (median = 7.92). Figure 1 shows the TL distribution (before logarithmic transformation) of participants by their parent-perceived family financial pressure in W1.

### 3.2. Associations between Parent-Perceived Family Financial Pressure and Their Level of Agreement on Stressors

Table 2 shows the results of regression models between parent-perceived family financial pressure and their level of agreement on each specific stressor item at the family, personal, and child level. Compared to families low in financial pressure, parents who perceived high family financial pressure faced more stressors (β = 1.38, *p* < 0.001). Specifically, they were more likely to agree on statements concerning child-level stressors including “When my child is sick, I am worried that I do not have the ability to afford his/her medical expenses” (β = 0.91, *p* = 0.003), “I do not have financial ability to afford my child’s educational expenses including tuition, book cost, and tutorial cost etc” (β = 1.19, *p* < 0.001), and “ I do not have financial ability to buy my child what he/she wants” (β = 0.98, *p* = 0.001). They were also more likely to agree on “Family financial burden is huge” (β = 1.19, *p* < 0.001), “I am dissatisfied with my financial condition” (β = 0.99, *p* < 0.001), and “Financial problems often worry me” (β = 1.35, *p* < 0.001). Similar patterns were found when comparing between parents who perceived population-equivalent family financial pressure and those who perceived low family financial pressure, although the differences in level of agreement on stressors between these two groups were smaller.

### 3.3. Effect of Early-Life Exposure to Family Financial Pressure on Telomere Length in Early Adolescence

Table 3 shows the results of the regression models between early-life exposure to family financial pressure and subsequent TL in early adolescence in the overall sample and by gender and family income status. When early-life exposure to family financial pressure was tested as a categorical variable, compared to the low family financial pressure group, participants in the high family financial pressure group had shorter telomeres (β = −0.61, *p* = 0.042). Results remained unchanged when early-life exposure to family financial pressure was tested as a continuous variable.

Notably, while TL showed significant associations with family financial pressure group membership, it did not significantly differ between gender and across family income levels. On the other hand, when the analyses were repeated within the girl group, early-life exposure to high family financial pressure had significant effects on TL (β = −0.97, *p* = 0.036), but such effects were not found in boys. Similarly, within the average-to-high family income group, participants exposed to high family financial pressure had significantly shorter TL when compared to those exposed to low family financial pressure (β = −0.72, *p* = 0.042), but these association patterns were not found in other family income groups.

## 4. Discussion

This study examined the effect of early-life exposure to family financial pressure on TL in early adolescence. We found that participants exposed to high family financial pressure, when compared to those exposed to low family financial pressure group, had significantly shorter TL, and such differences were particularly strong for those living in high-income families. Our study can thus extend previous research by demonstrating the cellular impact of mild family stressors. Results from this study add to the growing body of evidence on cellular aging which may accelerate the progression of disease due to early-life exposure to family stressors [15,16,26].

In this study, TL was found to be sensitive to the impact of early-life exposure to family financial pressure. While the majority of research on the relationship between family environment and TL has focused on extreme adverse events such as family violence and maltreatment experiences [16,27], there has been a growing body of research on moderate-intensity stressors such as disrupted family relationships and routine conflicts and arguments [15,26]. Our findings are in line with these recent studies showing that early-life exposure to mild family stressors including family financial pressure can negatively affect long-term health at the cellular level. As the family stress model indicates, dealing with financial hardship can trigger negative moods and poor family interactions such as parental psychological distress, family conflicts, and maladaptive parenting practices [6,7]. These family-level stressors have been found to be associated with telomere shortening [15]. Hence, overall evidence suggests that telomere is responsive and sensitive to the impact of early-life stressors.

One plausible biological pathway underlying the association between family financial pressure and shorter telomeres is the elevated chronic inflammatory responses. While inflammation is a protective physiological response against harmful stimuli such as pathogens, cellular injury, and irritants [28,29,30], dysregulated chronic inflammation can lead to local and systemic damages. Data from epidemiological and animal studies have shown an association between early adverse experiences and chronic inflammatory conditions [1,17,18,19,20,31]. Acute stress exposure might enhance the release of circulating pro-inflammatory cytokines such as interleukin (IL)-6, thereby promoting the development of auto-inflammatory condition [31]. This elevated inflammatory burden might be a reason for the observation that adolescents exposed to high family financial pressure early in life could have shorter telomeres in this study.

Our subgroup analyses by family income level showed that the negative effect of early-life exposure to family financial pressure on TL was observed mainly among participants in the average-to-high family income group, and such effect was weaker among participants in the low family income group. This indicates that the major contributor to shorter telomeres in this study was early-life exposure to family financial pressure, particularly in high family SES environment. To better understand the stressor profile of parents who perceived high family financial pressure, we examined various stressors in the family, personal, and child category by parent-perceived family financial pressure group membership. We found that the main source of stress for parents who perceived high family financial pressure were child-level stressors such as worrying whether they were financially capable to afford children’s medical and educational expenses as well as to fulfil children’s materialistic desires and needs. Notably, we found that the degree of willingness to organize family activities such as travel, watching movies or other special events was comparable between parents in the high family financial pressure group and those in the low family financial pressure group. This suggests that the negative effect of early-life exposure to family financial pressure on TL may not be due to reduced family activities. Instead, the findings suggest that parents perceiving high family financial pressure tend to experience higher levels of negative emotions such as stress and worry. Since adult emotions can be observed and absorbed by children during routine interactions and activities, transfer of negative emotions from parents to children is possible [32] and may potentially contribute to the acceleration of shorter telomeres observed in the participants exposed to high family financial pressure in early life.

In addition to family income, we found that the effect of early-life exposure to family financial pressure on subsequent TL in early adolescence differed by gender. Specifically, shorter TL was observed only in girls exposed to high family financial pressure in early life. These findings suggest that girls could be more sensitive to parental mood swings and thus have higher vulnerability to the adverse effect of family stressors. Consistent with our observations, previous studies also observed gender differences in the impact of early-life stress on TL in adolescence [16,33,34,35]. Although the biological explanations for such gender differences in health outcomes remained inconclusive, future studies should be aware of these potential gender differences when assessing the biological impact of early-life adversity.

In this study, participants were longitudinally followed up for 5 years and hence we can delineate the long-term health effect of childhood exposure to family financial pressure at the cellular level. However, this study also has limitations. First, we did not collect data on financial pressure in W2, so we cannot ascertain the chronic nature of financial pressure or infer associations between financial pressure and concurrent physiological responses. Second, because of the small sample size, our findings might not be generalizable to other population groups. Lastly, biomarkers were collected and measured only in W2 and from the index child in each family unit. Future work should recruit a bigger sample, ideally involving the index child and siblings, with longitudinal measurement of biomarkers at multiple time points spanning from childhood to adolescence to draw a more affirmative conclusion about the interplay of family environment and genetic influences on telomere shortening.

## 5. Conclusions

Our findings extend the current literature by documenting the effect of early-life exposure to family financial pressure on TL in early adolescence. Specifically, we found that children with early-life exposure to family financial pressure had shorter telomeres in early adolescence, and this association was particularly strong in girls and those in the average-to-high family income group. The findings indicate that TL is reflective of the long-term health consequences of adverse exposures. Our results align with the growing body of evidence across a broad range of biomarkers, suggesting that the cellular impact of childhood exposure to family stressors can emerge in early adolescence. As TL has important implications for disease development and progression, children living with financially stressed parents may have a greater risk for poor health outcomes in future. Family-based prevention programs that offer parenting skill training, employment opportunities, and financial assistance are needed to help these parents tackle the dual burden of financial problems and childcare responsibilities, which in turn would benefit child development.

## Figures and Tables

**Table 1:** Sample characteristics.

		Parent-Perceived Family Financial Pressure in W1			
	Overall	Low	Population-Equivalent	High	*p*
Age, W1, Mean (SD)	13.18 (0.39)	13.19 (0.40)	13.20 (0.40)	13.16 (0.37)	0.943
Sex, N (%)					0.451
Boys	52 (56.52%)	17 (53.13%)	26 (63.41%)	9 (47.37%)	
Girls	40 (43.48%)	15 (46.88%)	15 (36.59%)	10 (52.63%)	
Parental marital status, W1, N (%)					0.050
Married	84 (91.30%)	32 (100.00%)	38 (92.68%)	14 (73.68%)	
Single/divorced	6 (6.52%)	0 (0.00%)	2 (4.88%)	4 (21.05%)	
Mother’s employment status, W1, N (%)					0.500
Full-time	43 (46.74%)	17 (53.13%)	16 (39.02%)	10 (52.63%)	
Part-time	13 (14.13%)	7 (21.88%)	4 (9.76%)	2 (10.53%)	
Unemployed	1 (1.09%)	0 (0.00%)	1 (2.44%)	0 (0.00%)	
Homemaker	29 (31.52%)	8 (25.00%)	16 (39.02%)	5 (26.32%)	
Father’s employment status, W1, N (%)					0.222
Full-time	86 (93.48%)	32 (100.00%)	38 (92.68%)	16 (84.21%)	
Part-time	1 (1.09%)	0 (0.00%)	0 (0.00%)	1 (5.26%)	
Unemployed	2 (2.17%)	0 (0.00%)	1 (2.44%)	1 (5.26%)	
Homemaker	0 (0.00%)	0 (0.00%)	0 (0.00%)	0 (0.00%)	
Family income level (USD), W1, Mean (SD)	5924.20 (3008.04)	7900.64 (2619.90)	5304.49 (2410.54)	3900.13 (2950.98)	<0.001
Telomere length (T/S ratio), W2, Median (IQR)	7.92 (4.52)	8.55 (4.78)	7.98 (3.87)	7.37 (4.47)	0.356

**Table 2:** Associations between parent-perceived family financial pressure and their level of agreement on stressors.

	Parent-Perceived Family Financial Pressure in W1					
	Low		Population-Equivalent ^$^		High ^$^	
	*β* (95% CI)	*p*	*β* (95% CI) ^#^	*p*	*β* (95% CI) ^#^	*p*
Overall stressors	Ref	-	0.65 (0.26, 1.04)	0.001	1.38 (0.87, 1.88)	<0.001
a. Family financial burden is huge.	Ref	-	0.53 (0.05, 1.01)	0.029	1.19 (0.56, 1.81)	<0.001
b. If it increases expenditure, we would stop having family activities such as travel, watching movies or other special events.	Ref	-	0.35 (−0.08, 0.78)	0.115	0.49 (−0.07, 1.05)	0.088
c. Financial issues often cause family conflicts and harm family relationship.	Ref	-	0.29 (−0.18, 0.76)	0.224	0.55 (−0.07, 1.16)	0.080
d. I am dissatisfied with my financial condition.	Ref	-	0.59 (0.17, 1.00)	0.006	0.99 (0.44, 1.53)	<0.001
e. Financial problems often worry me.	Ref	-	0.49 (0.12, 0.86)	0.010	1.35 (0.87, 1.84)	<0.001
f. I do not have financial ability to celebrate holidays and other special days.	Ref	-	0.10 (−0.32, 0.53)	0.630	0.52 (−0.03, 1.08)	0.066
g. When my child is sick, I am worried that I do not have the ability to afford his/her medical expenses.	Ref	-	0.23 (−0.23, 0.70)	0.331	0.91 (0.30, 1.51)	0.003
h. I do not have financial ability to buy my child what he/she wants.	Ref	-	0.69 (0.23, 1.15)	0.004	0.98 (0.38, 1.58)	0.001
i. I do not have financial ability to afford my child’s educational expenses including tuition, book cost, and tutorial cost etc.	Ref	-	0.59 (0.05, 1.12)	0.031	1.19 (0.50, 1.88)	<0.001

Note: ^#^ Adjusted for age, gender of the child, family income level, parental marital status, and employment status in W1, ^$^ Compared to those with low parent-perceived family financial pressure.

**Table 3:** Associations between early-life exposure to family financial pressure and telomere length in early adolescence.

	Telomere Length, W2	
Family Financial Pressure, W1	*β* (95% CI)	*p*
Overall		
As categorical variable, W1		
High	−0.61 (−1.19, −0.02)	0.042
Population-equivalent	−0.40 (−0.84, 0.04)	0.077
Low	Ref	-
As continuous variable, W1	−0.34 (−0.56, −0.11)	0.004
Sub-group analyses by sex		
Boys		
As categorical variable, W1		
High	−0.25 (−0.91, 0.40)	0.447
Population-equivalent	−0.16 (−0.93, 0.60)	0.675
Low	Ref	-
As continuous variable, W1	−0.28 (−0.59, 0.03)	0.079
Girls		
As categorical variable, W1		
High	−0.97 (−1.99, −0.05)	0.036
Population-equivalent	−0.58 (−1.04, −0.12)	0.014
Low	Ref	-
As continuous variable, W1	−0.48 (−0.90, −0.06)	0.026
Sub-group analyses by family income status		
Average-to-high family income		
As categorical variable, W1		
High	−0.72 (−1.43, −0.02)	0.043
Population-equivalent	−0.39 (−0.89, 0.11)	0.130
Low	Ref	-
As continuous variable, W1	−0.35 (−0.60, −0.10)	0.007
Low family income		
As categorical variable, W1		
High	−0.44 (−1.24, 0.36)	0.285
Population-equivalent	0.25 (−0.40, 0.90)	0.443
Low	Ref	-
As continuous variable, W1	−0.13 (−0.66, 0.41)	0.603

Note: Adjusted for age and gender of the child, family income level, parental marital status and employment status in W1.

## Data Availability

The data that support the findings of this study are available from the corresponding author upon reasonable request.
